# Do no harm: the impact of implementing cancer prevention strategies on other preventive health measures

**DOI:** 10.1186/s43058-024-00597-6

**Published:** 2024-05-22

**Authors:** Karen M. Emmons, Leslie Pelton-Cairns, Daniel A. Gundersen, Jennifer L. Cruz, Lynette Mascioli, Gina R. Kruse, For the ISCCCE Partnership

**Affiliations:** 1 Department of Social and Behavioral Science, Harvard TH Chan School of Public Health, 677 Huntington Avenue, Boston, MA 2115 USA; 2 The Massachusetts League of Community Health Centers, 40 Court Street, 10th Floor, Boston, MA 02108 USA; 3 The Dana-Farber Cancer Institute, 450 Brookline Avenue, Boston, MA 02215 USA; 4 Division of General Internal Medicine, Massachusetts General Hospital, Harvard Medical School, 100 Cambridge Street, 16th floor, Boston, MA 02114 USA

**Keywords:** Implementation, Cancer screening, Unintended consequences

## Abstract

**Background:**

Translational efforts to increase uptake of evidence-based practices typically look at those outcomes in isolation of their impact on other aspects of care delivery. If we are in fact to “do no harm”, we must consider the possible negative impact of improving use of one practice on other quality measures. Alternatively, a focus on one practice could lead to spread of effective strategies to other practices, which would be highly beneficial. We studied the impact of a colorectal cancer (CRC) screening initiative on delivery of other preventive care measures.

**Methods:**

We used an interrupted time series design with implementation year as the interruption point. The initiative was conducted between 2015 and 2020, with three staggered cohorts. Main outcomes were quality measures for colorectal cancer screening, cervical cancer screening, hypertension management, diabetes management, weight screening and follow-up, tobacco use screening and cessation treatment, and depression screening and follow-up.

**Results:**

The initiative was associated with an increase in CRC screening (OR = 1.67, *p* *≤* 0.01; average marginal effect = 12.2% points), and did not reduce performance on other quality measures in the year of CRC program implementation or a change in their respective secular trends.

**Conclusions:**

The initiative led to a clinically meaningful increase in CRC screening and was not associated with reductions in delivery of six other preventive services. Quality improvement (QI) initiatives typically approach implementation with an eye towards reducing unintended impact and leveraging existing staff and resources. Implementation research studies may benefit from considering how QI initiatives factor in the local context in implementation efforts.

Contributions to the literature

- This is one of the first papers to our knowledge that examines the impact of an implementation strategy promoting one EBI on other preventive health services. Since many implementation strategies are systems-level, it would have value to understand their impact on care delivered more broadly in the implementing system.
- This study adopts a systems-level view of prevention across a number of clinical quality targets, beyond impact on cancer-related outcomes, reflecting both health system and patient-level interests broadly.
- This study includes a number of federally-qualified community health centers operating in real world settings, and thus provides a realistic view of the outcomes of interest.

## Introduction

‘First, do no harm’ has been the cornerstone of medicine since the 5th century BC, when Hippocrates noted that physicians have two objectives with regard to disease– to do good, and to do no harm. The modern interpretation of this principle is that doctors should help their patients as much as they can by recommending tests or treatments for which the potential benefits outweigh the risks of harm [1]. A key part of preventing harm is to ensure that one treatment is not considered in isolation from others, as in the case with avoiding drug interactions and selection of drugs that consider comorbid conditions. For example, the use of bupropion by a patient who smokes and has diabetes could both promote cessation and prevent weight gain that could exacerbate their diabetes. An example of the potential benefits of treating one disease on other health outcomes is the use of semaglutide, which could both improve blood sugar control and promote weight loss in patients with obesity and diabetes. The ‘do no harm’ principle is often viewed through both ethical and patient safety lenses, which have received considerable attention over the past few decades. This principle is further exemplified by the IOM report, Crossing the Quality Chasm [2], which emphasizes the importance of healthcare institutions avoiding harm to patients from care that is intended to help them. Equally important is considering when treatments may have synergistic benefits for multiple disease outcomes.

Translational science has as a primary goal the hastening of the scientific process required to develop and deliver treatments that improve people’s lives, or as Hippocrates notes, that do good [3, 4]. The fields of implementation science and quality improvement sit on the translational continuum and focus on increasing uptake of evidence-based clinical services and practices, so that all populations can benefit regardless of where they receive care. Implicit within this is an assumption that effectively putting an evidence-based practice into place will do good—that it will increase use of a beneficial approach, and as a result health will be improved. Given the focus on use of evidence-based programs, many implementation science studies examine outcomes at the institutional level, rather than for the individual patient. The research question is often about which strategies promote uptake of evidence-based interventions, under what circumstances and in what context. Thus, the risk to individual participants is often minimal, approximating the same level of risk associated with the delivery of routine clinical care [5].

Fiscella, et al. [6] have argued that data safety monitoring boards (DSMB) may have limited applicability for implementation studies, as NIH does not generally require DSMBs for minimal risk trials. Further, typical implementation study features (e.g. use of mixed methods, collection of effectiveness data from the EHR) and outcomes (e.g., acceptability, adoption, appropriateness, feasibility, reach) don’t align well with typical DSMB stopping rules. The authors note that any harm caused by implementation studies would more likely be at the organizational level, particularly in terms of impacts on the workflow and workforce. They recommend that investigators begin to collect and monitor data addressing potential, unintended consequences for organizational-level outcomes. This is an extremely important observation, and it is worth noting that studies on the ethics of evidence-based practice rarely mention the importance of evaluating impact on system-level delivery of care [7].

There is a significant gap in our knowledge on the impact of strategies to implement evidence-based interventions on other organizational or population-level outcomes. This is particularly a concern in under-resourced settings, where there are finite staffing resources and new implementation efforts often are assigned to those already responsible for significant clinical activity. Federally-qualified community health centers (FQHCs), which provide care for over 30 million people nationally, do an excellent job delivering evidence-based health care, despite the fact that they typically operate in communities with significant resource constraints and provide care to all who come. We know little about how to ensure that new activities are absorbed into these delivery systems while allowing other clinical priorities to be maintained. It seems plausible, for example, that the implementation of an effort to increase one cancer screening test in a FQHC’s adult primary care practice could lead the clinical staff to focus heavily on that test, at the expense of other routine prevention activities. Thus, the uptake of new implementation activities may lead to diverted attention and resources from other clinical services. Alternatively, an implementation strategy focused on one practice could lead to the spread of effective strategies to other clinical services, and contact with patients about one screening need could re-engage them in care to address other health care needs. To our knowledge there have been no studies empirically examining such impacts.

The purpose of this paper is to evaluate the impact of implementation of a quality improvement initiative to increase colon cancer screening among 14 community health centers serving low-income populations on the delivery of other preventive care or chronic disease management activities. The initiative was led by the Massachusetts League of Community Health Centers (Mass League), which serves as the primary care association for FQHCs in Massachusetts. The initiative launched in December of 2015 with the first of three staggered cohorts. The third cohort graduated from the initiative in June of 2020. This study frames the implementation from the perspective of FQHC operations, and how the course of integration of new clinical initiatives and their impacts are managed.

## Methods

This study was reviewed by the Harvard Longwood IRB and received a determination that it was not human subjects research. The data was pulled for analysis in 2022.

### Colorectal Cancer Screening (CRCS) Quality Improvement (QI) initiative

The implementation strategies used in the CRCS QI Initiative included a Learning Collaborative based on the Institute for Healthcare Improvement’s Model for Improvement, and improvement coaching between learning sessions. Quarterly learning sessions included content focused on evidence-based implementation strategies shown to improve colorectal cancer screening, education by faculty experts in colorectal cancer screening, and education about the Model for Improvement and other quality improvement tools and techniques. Another critical feature of the Learning Collaborative intervention was the sharing of best practices among participants, often focused on clinic operations, which took place during learning sessions. Health centers used action periods between sessions to run Plan-Do-Study-Act (PDSA) cycles, monitored CRC screening rates quarterly, and received improvement coaching following report submissions. Participating health center teams typically included a project lead from the quality department, a clinician champion, and a lead medical assistant, with other staff supporting, as needed. The three cohorts launched at different times between 2015 and 2020 and some cohorts overlapped, with the shortest participation lasting 23 months and the longest participation lasting 36 months. Health centers received $20,000 per year to participate in the initiative, to be used flexibly to support their participation.

The implementation goal for the Learning Collaborative was to cause as little disruption as possible resulting from requirements and interactions with health centers. Health centers were offered a Driver Diagram, mapping out implementation strategies that they could choose to use or not, based on their context. Successes and failures with PDSAs were shared across the cohorts to help other FQHCs select and use the implementation strategies. Existing workflows and staffing were taken into account and modified in ways that could be sustained over time without funding. Time away from clinic was minimized for the provider champions, and meetings were scheduled at the convenience of health center teams. The primary ethical considerations were associated with not inadvertently disrupting the overall care delivery process and ensuring that the implementation strategies were sustainable. FQHCs were given considerable flexibility in their approach to ensure that it fit and long-term usability within the care delivery setting.

### Study sample

This study examines data from 10 of the 14 community health centers that participated in the CRC Screening initiative. Four FQHCs were excluded as one FQHC system had data migration issues, two participating sites report their data to HRSA under one FQHC license and thus their data could not be disaggregated, and one FQHC system with data reporting under one FQHC license had only one site elect to participate due to staffing issues at the other sites, and thus the participating site’s data could not be isolated. Data were drawn from each participating FQHC’s data that is reported annually to Health Resources and Services Administration (HRSA) as part of the Uniform Data System (UDS). Data were available for different years depending on the preventive service or chronic disease management outcome and the UDS reporting requirement at that time. We constructed a data set of FQHC-level data from 2009 to 2020, with data available starting in 2012 for colorectal cancer screening; 2009 for cervical cancer screening, hypertension with BP in control, and diabetics with HBA1C not in control; 2011 for weight assessment and follow-up, and tobacco use screening and cessation counseling; and 2014 for depression screening and follow-up. Of the 10 health centers in the sample, there were three FQHCs in Cohorts 1 and 2, and 4 FQHCs in Cohort 3.

### Measures

Because our interest was in whether or not implementation of the CRC screening effort impacted on delivery of other preventive care or chronic disease management services, we examined clinical quality measures for these services. The specific measures utilized are those required for routine data collection by HRSA, through the UDS, and detailed in Table 1. All outcomes are reported as the proportion of the eligible population that received the respective screenings or met the criteria for chronic disease management. Note that because breast cancer screening was added as a UDS measure in 2020, it was not assessed during the CRC initiative implementation period for all of the cohorts, and thus is not included. Further, although CRC screening eligibility was recently changed to age 45 by the USPSTF, during the analytic period the recommendation and corresponding UDS measure included age 50 and older.

**Table 1:** UDS measures of preventive care services and chronic disease management

***Preventive Service***	***Measure***	
Colorectal Cancer Screening	Percentage of adults 50–75 years of age who had appropriate screening for colorectal cancer	
Cervical cancer screening	Percentage of women 21–64 years of age who were screened for cervical cancer using either of the following criteria: (1) Women aged 21–64 who had cervical cytology performed every 3 years; or (2) Women age 30–64 who had cervical cytology/human papillomavirus every 5 years	
Weight Assessment and Follow-Up	Percentage of patients aged 18 years and older with BMI documented during the most recent visit or within the previous 12 months to that visit; and when the BMI is outside of normal parameters, a follow-up plan is documented during the visit or during the previous 12 months of that visit	
Tobacco Use Screening and Cessation Intervention	Percentage of patients aged 18 years and older who were screened for tobacco use one or more times within 24 months and who received cessation counseling intervention if defined as using tobacco	
***Chronic Disease Management***	***Measure***	
Patients with hypertension with BP in control	Percentage of patients 18–85 years of age who had a diagnosis of hypertension overlapping the measurement period and whose most recent blood pressure (BP) was adequately controlled (less than 140/90 mmHg) during the measurement period	
Patients with diabetes with HbA1C not in control (inverse coded for the purpose of analysis)	Percentage of patients 18–75 years of age with diabetes who had hemoglobin A1c (HbA1c) greater than 9.0% during the measurement period Numerator: Patients whose most recent HbA1c level performed during the measurement year was greater than 9.0% or patients who had no test conducted during the measurement period	
Depression Screening and Follow-Up	Percentage of patients aged 12 years and older screened for depression on the date of the visit or 14 days prior to the visit using an age-appropriate standardized depression screening tool and, if positive, had a follow-up plan documented on the date of the visit	

We also invited FQHC staff members at each FQHC to participate in an interview in which the FQHC’s data was reviewed and feedback was sought to explore any contextual factors that were important to understand related to data interpretation. Four FQHCs had a staff member agree to participate. Where possible, participants were part of the CRCS initiative, although in some cases those staff had were no longer at the FQHC and the current quality improvement lead participated.

### Statistical analysis

We conducted an interrupted time series analysis using the CRC screening program’s implementation year for the respective cohorts as the interruption point for all outcomes. To evaluate both short and long-term implementation impact, we estimated change in the preventive screening or chronic disease management rate in the year of implementation, as well as changes in their trends over time by fitting separate segmented beta regression models for each outcome. Each model was fit using logit link and included dummy variables for FQHCs to control for factors that vary across FQHCs but are time-invariant within FQHCs. P-values were calculated based on heteroscedastic and autocorrelation consistent standard errors and adjusted using the Benjamini-Yekutieli method to control the false discovery rate [8]. To address data at the extremes of the probability range we transformed each outcome using the method described by Smithson and Verkulien [9]. All analyses were conducted in R and parameters estimated using the betareg package with the sandwich package used to produce heteroscedastic and autocorrelation consistent standard errors [10–13].

## Results

The model-based results for each outcome are presented in Table 2. Overall, there was a significant and clinically meaningful increase in the rate of colorectal cancer screening in the year of implementation (OR = 1.67, *P* = 0.007), corresponding to an average marginal effect of 12.2% points. There was no change in the trend following implementation, which remained flat (OR = 1.01, *P* = 0.874). Cervical cancer screening had a declining trend in screening rate prior to and after CRC screening implementation (trend: OR = 0.92, *P* = 0.008; Change in trend: OR = 0.95, *P* = 1.0); however, there was a significant increase in cervical cancer screening the year of CRC screening program implementation (OR = 1.61, *P* = 0.049; average marginal effect = 11.0% points). No other screening or chronic disease management outcome had a significant change in the year of CRC program implementation or a change in their respective secular trends.

**Table 2:** Estimated trend, change in trend, and change in year of  colorectal cancer screening implementation by outcome

	log-OR			OR		Se	|t-value|	BY^1^ *p*-values
*Colorectal cancer screening*								
Trend	-0.06			0.94		0.049	1.17	1.00
Change in trend	0.01			1.01		0.057	0.17	1.000
Change in year of implementation	0.51			1.67		0.124	4.14	<0.01
*Cervical cancer screening*								
Trend	-0.09			0.92		0.023	3.87	< 0.01
Change in trend	-0.05			0.95		0.044	1.19	1.00
Change in year of implementation	0.48			1.61		0.155	3.09	< 0.05
*Weight Assessment and Follow-Up*								
Trend	0.06			1.06		0.036	1.67	0.75
Change in trend	-0.13			0.88		0.071	1.82	0.62
Change in year of implementation	0.27			1.31		0.205	1.32	1.00
*Tobacco Use Screening and Treatment*								
Trend	-0.03			0.97		0.135	0.23	1.00
Change in trend	-0.02			0.98		0.153	0.15	1.00
Change in year of implementation	0.07			1.07		0.250	0.26	1.00
*Diabetes with HBA1C in control*								
Trend	0.06			1.06		0.016	3.39	< 0.03
Change in trend	0.04			1.05		0.028	1.58	0.82
Change in year of implementation	-0.12			0.88		0.080	1.53	0.82
*Hypertension with BP in control*								
Trend	-0.03			0.97		0.018	1.84	0.62
Change in trend	-0.05			0.95		0.049	1.09	1.00

The qualitative interviews with FQHC staff provided helpful contextual information related to the study findings. They shared that it is common for health centers to focus on improving multiple measures throughout a program year. Staff were not surprised that the CRC initiative did not negatively impact on other quality measures. They also noted a range of external factors affecting their systems that would be more likely to negatively impact implementation outcomes than would an improvement initiative. Such factors included the impact of changes in leadership and electronic medical record systems, as well as high staff turnover and vacancies in key positions. FQHC staff also pointed to the general experience of declines in quality measures when regulators, in this case HRSA, make even small changes to measure definitions.

## Discussion

The goal of this study was to explore whether or not implementation of a learning collaborative to increase CRC screening across a number of FQHCs was associated with an increase or decrease in delivery of other preventive or chronic disease management services the year of CRC program implementation or by changing their respective secular trends (e.g. by creating or accelerating a declining screening or chronic disease management rate). Using annual FQHC-level data, we found that the CRC screening initiative led to a clinically meaningful increase in CRC screening and was not associated with reductions in delivery of other preventive services overall. There was an increase found in cervical cancer screening the year of implementation of the CRC screening initiative. This could reflect increased attention to cancer screening overall and may also be attributed to another training and technical assistance initiative offered by Mass League to increase cervical cancer screening rates. Five of the 10 FQHCs participated in this initiative, two after completion of the CRC initiative, and three at the same time. The baseline cervical cancer screening rates for this group of FQHCs were lower than for the five FQHCs that did not participate in the cervical cancer screening initiative.

These findings are especially impressive given the environmental conditions in which FQHCs in Massachusetts were operating under during the study period. In 2016, there was a $30 M reduction in Health Safety Net coverage for uninsured and underinsured patients, which placed additional financial burdens on health centers that are required to serve patients regardless of ability to pay. Further, by 2017 competition for healthcare workers, especially primary care providers, was intensifying, in part because of the significantly lower salaries paid to physicians and nurses in FQHC vs. hospital and private practice settings. The pressure on staffing only grew during the pandemic.

There are some limitations to this work that should be noted. First, we utilized UDS data, which offers a high level, systems view of changes over time. Although there can be issues with quality of system level data, the Mass League works closely with FQHCs in Massachusetts to support data quality assurance. Further, the multi-year evaluation used here minimizes the impact of short-term data issues that are occasionally encountered, in particular using an interrupted time series design whereby FQHCs serve as their own control. Second, the CRC Screening Initiative was not the only focused effort going on in these FQHCs. In 2017, there was a significant focus on building capacity to address substance use services related to the Opioid Epidemic. In 2017–2020 a cervical cancer screening initiative was underway, as mentioned above. In 2019, HRSA began a major push on diabetes management. Further, there has been a significant transformation effort underway in Massachusetts’ Medicaid Program to increase delivery of value-based care through accountable care organizations. However, these types of activities will always be present in a dynamic health care environment, and it is simply not possible to hold all things constant. The analyses conducted reflect implementation efforts in the context of real-world circumstances, which is exactly what we hoped to capture. There were some FQHCs invited to participate that declined due to staffing issues. However, most FQHCs face at least some staffing issues, including those that chose to participate. Finally, the participating FQHCs were all located in one state; however, they did include sites serving a wide range of geographies, ranging from large metropolitan cities to rural settings.

The demonstration that implementation of a CRC screening initiative did not negatively impact other services is excellent news. These findings were unsurprising to our community partners, who approach implementation with an eye towards minimizing unintended impact. We cannot assume that the findings will replicate without using the implementation approach studied here. It is also important to note that this quality improvement initiative was fully led by FQHC staff and their partners, and not researchers. Implementation research studies should be attentive to the adequacy of the support that they provide to participating FQHCs, and that the research burden is minimized. We believe that it is important for the field to begin to routinely evaluate the impact of focused implementations on care delivery more broadly, especially as the pressures associated with the current fiscal and political climate may put further stress on community health systems.

## Conclusions

The initiative led to a clinically meaningful increase in CRC screening and was not associated with reductions in delivery of six other preventive services. Quality improvement (QI) initiatives typically approach implementation with an eye towards reducing unintended impact and leveraging existing staff and resources. Implementation research studies may benefit from considering how QI initiatives factor in the local context in implementation efforts.

## Data Availability

Data were drawn from each participating FQHC’s data that is reported annually to Health Resources and Services Administration (HRSA) as part of the Uniform Data System (UDS) (https://data.hrsa.gov/tools/data-reporting/program-data).

## Abbreviations

DSMB: Data safety monitoring boards
FQHC: Community health center
CRC: Colorectal Cancer
CRCS: Colorectal Cancer Screening
QI: Quality improvement
Mass League: Massachusetts League of Community Health Centers
PDSA: Plan-Do-Study-Act
EBIs: Evidence based interventions
HRSA: Health Resources and Services Administration
UDS: Uniform Data System
